# Supplementary material for: Defining Optimal Conditions for Tumor Extracellular Vesicle DNA Extraction for Mutation Profiling
Source: Cancers (Basel). 2022 Jul 2;14(13):3258. doi: 10.3390/cancers14133258 (PMC9265681; doi:10.3390/cancers14133258)
Supplement: Supplementary file 1 [file cancers-14-03258-s001.zip › cancers-1761083- File S1.pdf]

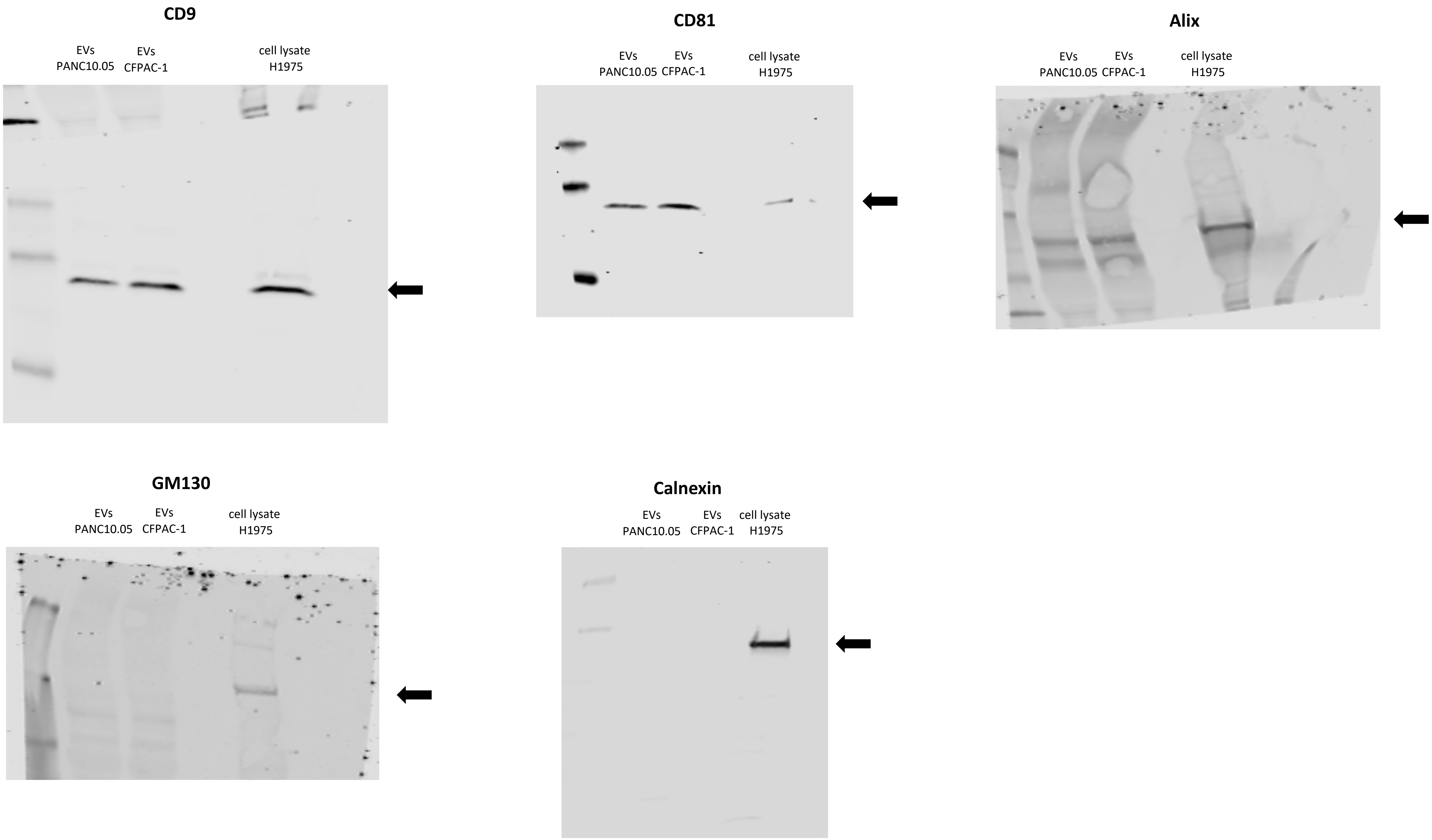
CD9
EVs PANC10.05
EVs CFPAC-1
cell lysate H1975
CD81
EVs PANC10.05
EVs CFPAC-1
cell lysate H1975
Alix
EVs PANC10.05
EVs CFPAC-1
cell lysate H1975
GM130
EVs PANC10.05
EVs CFPAC-1
cell lysate H1975
Calnexin
EVs PANC10.05
EVs CFPAC-1
cell lysate H1975
