# Supplementary material for: Defining Optimal Conditions for Tumor Extracellular Vesicle DNA Extraction for Mutation Profiling
Source: Cancers (Basel). 2022 Jul 2;14(13):3258. doi: 10.3390/cancers14133258 (PMC9265681; doi:10.3390/cancers14133258)
Supplement: Supplementary file 1 [file cancers-14-03258-s001.zip › cancers-1761083-supplementary.pdf]

cancers

Supplementary Material: Defining Optimal Conditions for Tumor Extracellular Vesicle DNA Extraction for Mutation Profiling

Julia Elzanowska, Laura Berrocal, Beatriz García-Peláez, Marta Vives-Usano, Beatriz Passos Sebo, Joana Maia, Silvia Batista, Jaakko Teppo, Markku Varjosalo, Maria Carolina Strano Moraes, Miguel Ángel Molina-Vila and Bruno Costa-Silva

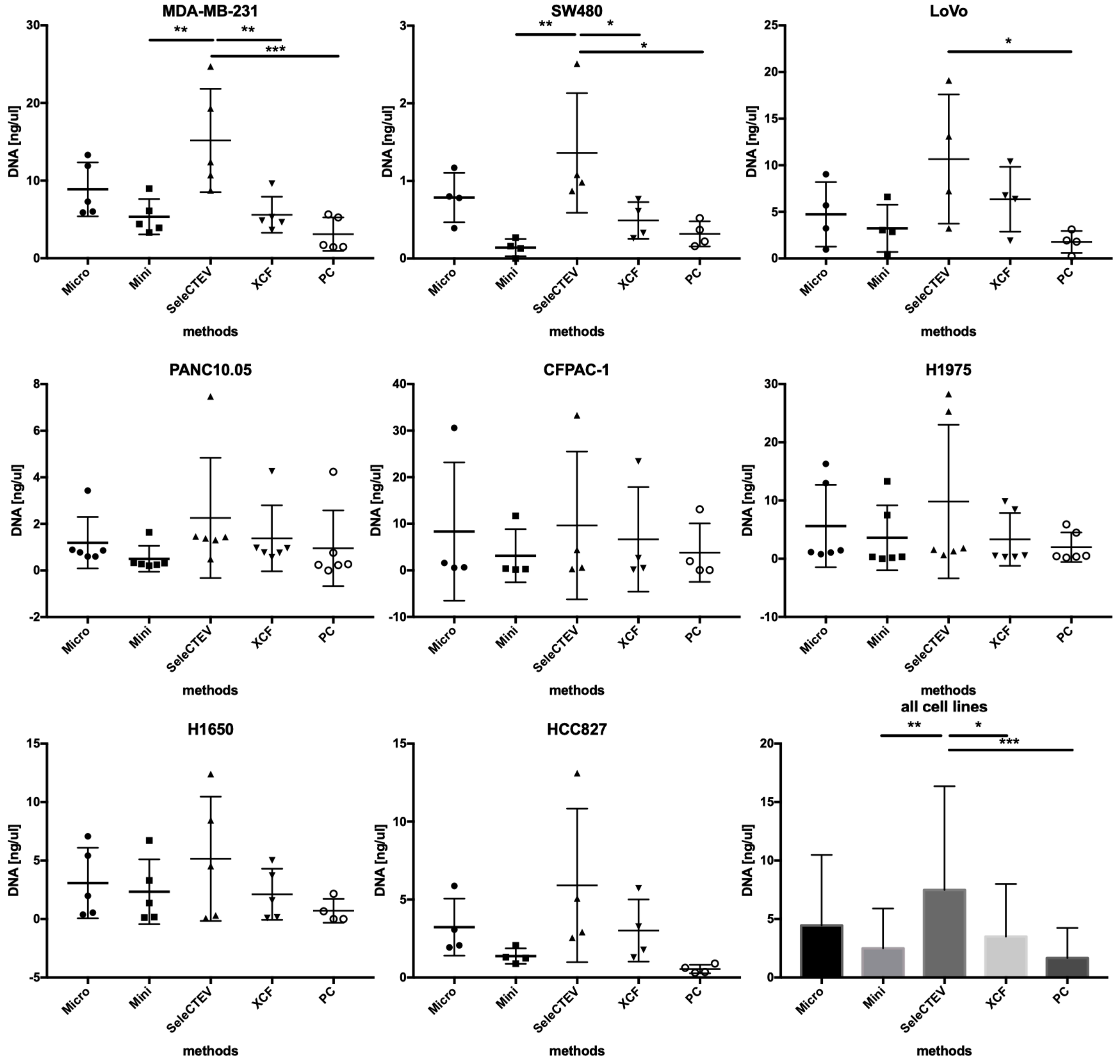

Figure S1. Comparison of EV-DNA abundance between different DNA extraction methods. Qubit DNA quantifications in EV-DNA samples extracted from a panel of cancer cell lines (MDA-MB-231, SW480, LoVo, PANC10.05, CFPAC-1, H1975, H1650 and HCC827) using QIAamp DNA Micro Kit (Micro), QIAamp DNA Mini Kit (Mini), SeleCTEV Exosomal DNA Kit (SeleCTEV), XCF Exosomal DNA Kit (XCF) and phenol chloroform. EV-DNA levels are presented as ng of DNA/µl. All data is shown as the mean values ±SD from independent measurements. P-values were calculated using one-way ANOVA with Tukey's post-test; *** $P < 0.001$, ** $P < 0.01$, * $P < 0.05$.

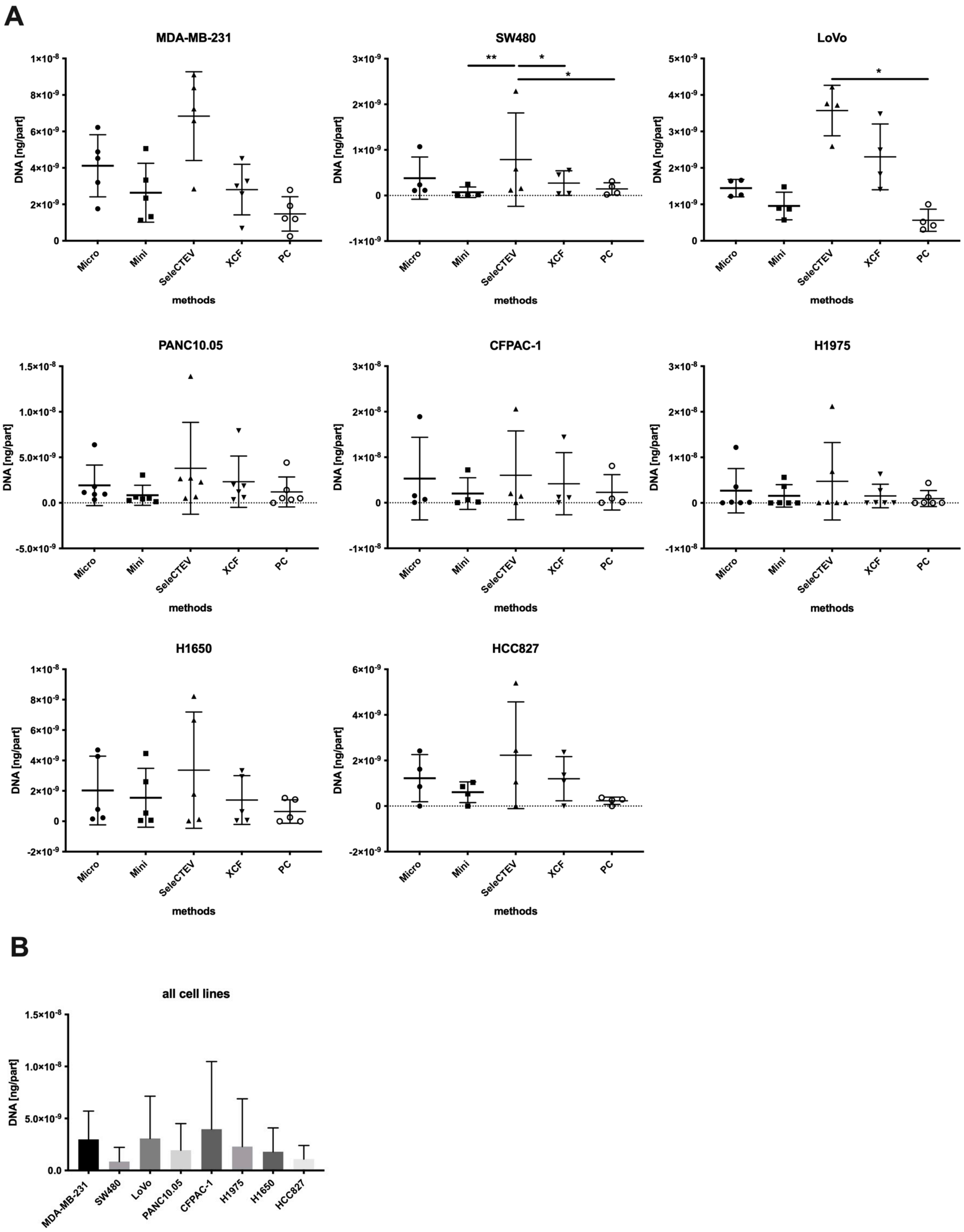

Figure S2. Comparison of EV-DNA abundance per particle. Qubit DNA quantifications in EV-DNA samples extracted from a panel of cancer cell lines (MDA-MB-231, SW480, LoVo, PANC10.05, CFPAC-1, H1975, H1650 and HCC827) using QIAamp DNA Micro Kit (Micro), QIAamp DNA Mini Kit (Mini), SeleCTEV Exosomal DNA Kit (SeleCTEV), XCF Exosomal DNA Kit (XCF) and phenol chloroform. EV-DNA levels were compared between different extraction methods (**A**) and analyzed cancer cell lines (**B**). EV-DNA levels are presented as levels of DNA normalized by the number of particles in each EV sample. All data is shown as the mean values ±SD from independent measurements. P-values were calculated using one-way ANOVA with Tukey's post-test; *** $P < 0.001$, ** $P < 0.01$, * $P < 0.05$.

Table S1. Number of cells seeded at the initiation of culture for conditioning.

Cell line	Number of cells/plate
MDA-MB-231	1.25×10^6
SW480	1.0×10^6
LoVo	1.5×10^6
PANC10.05	2.0×10^6
CFPAC-1	1.25×10^6
H1975	1.0×10^6
H1650	1.5×10^6
HCC827	1×10^6

Table S2. Cell lines used as positive and negative controls for TaqMan.

Gene	Positive control	Negative control
EGFR		
**Exon 19**	PC9	BxPC-3
**Exon 21**	11-18	BxPC-3
**T790M**	H1975	BxPC-3
KRAS		
**Exon 2**	H23, DLD1	BxPC-3
**Exon 3**	H460	BxPC-3
BRAF		
**Exon 15**	HT29	BxPC-3

Table S3. The clinical characteristics of patients.

Patient characteristics				Mutations	
**Patient**	**Tumor type**	**Sex**	**Age**	**Gene**	**Position**
#1	Melanoma	F	N/A	*BRAF*	p.V600E
#2	Colorectal cancer	M	84	*KRAS*	p.G12V
#3	Lung cancer	F	68	*KRAS*	p.G12C
#4	Lung cancer	M	81	*KRAS*	p.G12D
#5	Cervical cancer	F	43	*KRAS*	p.G12V
#6	Lung cancer	M	66	*BRAF*	p.V600E

Table S4. Technical aspects of patients' blood sample collection and processing.

Patient	Tumor type	Time of day of collection [hour]	Blood collection volume [mL]	First tube discard	Hemolysis *	Transport and sample processing
#1	Melanoma	09:57	28	No	No	N/A; samples were kept in an upright position and processed directly after collection
#2	Colorectal cancer	12:29	21	No	No	
#3	Lung cancer	16:32	28	No	No	
#4	Lung cancer	11:36	21	No	No	
#5	Cervical cancer	12:46	21	No	No	
#6	Lung cancer	10:02	28	No	No	

* Plasma samples were visually inspected, and no samples had obvious signs of hemolysis.visual inspection based on color chart for detection of hemolysis.

Table S5. List of primary and secondary antibodies used for Western blotting.

Primary Antibodies			
**Antibody**	**Manufacturer**	**Catalog no**	**Dilution/concentration**
CD81	Santa Cruz Biotechnology	sc-166029	1:50
CD9	Cell Signaling	#13174	1:1000
Alix	Sigma Aldrich	SAB4200476-200UL	1.25 ug/mL
Calnexin	Abcam	ab22595	1:2000
GM130	Abcam	ab52649	1:1000
**Secondary Antibodies**			
**Antibody**	**Manufacturer**	**Catalog no**	**Dilution**
Goat anti-rabbit IgG IRDye 800CW	LI-COR Biosciences	926-32211	1:5000
Goat anti-mouse IgG IRDye 800CW	LI-COR Biosciences	926-32210	1:5000

Table S6. Primers and probes sequences used in TaqMan.

Gene	Name	Sequence
EGFR	19_F_TAQM	5′ TGAGAAAGTTAAAATTCCCGTCG 3′
	19_R_TAQM	5′ GAAACTCACATCGAGGATTTCCTT 3′
	EGFR-T790F	5′ CCTCACCTCCACCGTGCA 3′
	EGFR-T790R	5′ AGGCAGCCGAAGGGCA 3′
	EGFR-858F	5′ AACACCGCAGCATGTCAAGA 3′
	EGFR-861R	5′ TGCCTCCTTCTGCATGGTATTC 3′
	19_TAQM_WT2	5′ VIC-TCAAGGAATTAAGAGAAG-MGB 3′
	19_TAQM_E746-A750del	5′ FAM- TCAAAACATCTCCGAAAG –MGB 3′
	19_TAQM_E746-A750*del	5′ FAM- TCAAGACATCTCCGAAAG -MGB 3′
	19_TAQM_L747-T751del	5′ FAM- TCAAGGAATCTCCGAAAG -MGB 3′
	19_TAQM_E746-T751>A	5′ FAM- TCAAGGCATCTCCGAAA -MGB 3′
	19_TAQM_L747-P753>S	5′ FAM- AGGAATCGAAAGCCAA -MGB 3′
	19_TAQM_ L747-S752del	5′ FAM-TCAAGGAACCGAAAGC-MGB 3′
	19_TAQM_ E746-S752>V	5′ FAM- TCAAGGTTCCGAAAGC-MGB 3′
	19_TAQM _L747-T751>P	5′ FAM- AAGGAACCATCTCCGAA-MGB 3′
	19_TAQM _L747-A750>P	5′ FAM- CAAGGAACCAACATCT -MGB 3′
	E21_858-FAM	5′ FAM-ATTTTGGGCGGGCCAAAC-MGB 3′
	E21_861-FAM	5′ FAM-TGGCCAAACAGCTGGGTG-MGB 3′
	E21_858-VIC	5′ VIC-ATTTTGGGCTGGCCAAAC-MGB 3′
	E20_T790M-FAM	5′ FAM-CTCATCATGCAGCTCATG-MGB 3′
	E20_T790M-VIC	5′ VIC- CTCATCACGCAGCTCATG -MGB 3′
	PNA_ex19	5′ AGATGTTGCTTCTCTTA 3′
	PNA_ex21	5′ AGTTTGGCCAGCCCA 3′
	PNA_ex20	5′ TCATCACGCAGCTC 3′
KRAS	KRAS_TAQM_12F	5′ GCCTGCTGAAAATGACTGAA 3′
	KRAS_TAQM_12R	5′ TATCGTCAAGGCACTCTTGC 3′
	KRAS_TAQM_61F	5′ TGGAGAAACCTGTCTCTTGGA 3′
	KRAS_TAQM_61R	5′ TCCTCATGTACTGGTCCCTCA 3′
	KRAS_12_WT	5′ VIC - ACGCCACCAGCTCCA – MGB 3′

	KRAS_61_WT	5′ VIC – CAGGTCAAGAGGAG - MGB 3′
	KRAS_G12C	5′ FAM-ACGCCACAAGCTCCA-MGB 3′
	KRAS_G12D	5′ FAM-ACGCCATCAGCTCCA-MGB 3′
	KRAS_G12V	5′ FAM-ACGCCAACAGCTCCA-MGB 3′
	KRAS_G12A	5′ FAM-ACGCCAGCAGCTCCA-MGB 3′
	KRAS_G12S	5′ FAM-ACGCCACTAGCTCCA-MGB 3′
	KRAS_G12R	5′ FAM-ACGCCACGAGCTCCA-MGB 3′
	KRAS_G13D	5′ FAM-ACGTCACCAGCTCCA –MGB 3′
	KRAS_G13C	5′ FAM-ACGCAACCAGCTCCA-MGB 3′
	KRAS_Q61H_1	5′ FAM-CAGGTCATGAGGAG-MGB 3′
	KRAS_Q61H_2	5′ FAM-CAGGTCACGAGGAG-MGB 3′
	KRAS_Q61L	5′ FAM-CAGGTCTAGAGGAG-MGB 3′
	PNA_KRAS_EX2R	5′ CTACGCCACCAGCTC 3′
	PNA_KRAS_EX3R	5′ CTCCTCTTGACCTGC 3′
BRAF	BRAF_TAQ_F	5′ CTACTGTTTTCCTTTACTTACTACACCTCAGA 3′
	BRAF_TAQ_R	5′ ATCCAGACAACTGTTCAAACTGATG 3′
	BRAF_F	5′ TGCTTGCTCTGATAGGAAAATG 3′
	BRAF_R	5′ TCAGTGGAAAAATAGCCTCAA 3′
	BRAF_V600E_FAM	5′ FAM-TAGCTACAGAGAAATC-MGB 3′
	BRAF_V600E_VIC	5′ VIC-CTAGCTACAGTGAAATC-MGB 3′
	BRAF_V600K_FAM	5′ FAM-TAGCTACAAAGAAATC-MGB 3′

Table S7. Customized panel for NGS.

Gene	CNVs analysis	Exons
EGFR	**Y**	12, 18, 19, 20, 21
BRAF	**Y**	11, 15
MET	**Y**	13, 15 (splicing MET14)
ERBB2	**Y**	8, 17, 19, 20, 21
ALK	**N**	22, 23, 24, 25
ROS1	**N**	38
PIK3CA	**N**	10, 21
KRAS	**Y**	2, 3, 4
NRAS	**N**	2, 3, 4
FGFR1	**Y**	X
KIT	**N**	8, 9, 11, 13, 14, 17
PDGFRA	**N**	12, 18
TP53	**N**	4, 5, 6, 7, 8, 9
CDK4	**Y**	X
CDK6	**Y**	X
RCTOR	**Y**	X
IDH1	**N**	4
IDH2	**N**	4
STK11	**N**	2, 3, 4, 5, 6, 7, 8, 9
ERBB4	**N**	8, 17, 19, 20, 21

Table S8. Comparison of ng of DNA/mL of media in non-conditioned media and cell conditioned media.

Sample	Total volume of media [ml]	Total DNA [ng]	ng of DNA/ml of media
nCM1	270	12	0.04
nCM2	135	10	0.07
nCM3	135	4.4	0.03
CCM1	600	262	0.44
CCM2	600	506	0.84
CCM3	520	382	0.74

nCM - non-conditioned media; CCM - cell conditioned media.

Table S9. Coefficients of variation (CV) calculated for EV-DNA extraction methods.

		EV-DNA purification method				
	**Cell-line derived EVs**	**Micro**	**Mini**	**SeleCTEV**	**XCF**	**PC**
**Coefficient of variation (CV)**	MDA-MB-231	39.11%	42.69%	43.84%	41.33%	69.52%
	SW480	40.45%	79.33%	56.68%	48.25%	50.90%
	LoVo	73.15%	78.34%	64.98%	54.70%	66.58%
	PANC10.05	92.41%	110.84%	114.26%	102.62%	170.14%
	CFPAC-1	177.88%	181.90%	164.48%	168.28%	164.74%
	H1975	125.77%	154.72%	134.23%	136.49%	127.82%
	H1650	97.93%	118.38%	103.22%	103.38%	144.49%
	HCC827	56.63%	35.90%	83.33%	66.13%	50.89%
	**all cell lines**	135.67%	136.81%	118.40%	128.51%	153.99%
